# Supplementary material for: Environmental and Economic Assessment of Repairable Carbon-Fiber-Reinforced Polymers in Circular Economy Perspective
Source: Materials (Basel). 2022 Apr 20;15(9):2986. doi: 10.3390/ma15092986 (PMC9105246; doi:10.3390/ma15092986)
Supplement: Supplementary file 1 [file materials-15-02986-s001.zip › materials-1621277-supplementary.pdf]

Supplementary Materials

Contents

S1. Introduction **3**
S1.1 Life Cycle Assessment on composites 3

S2. Results **5**
S2.1 Normalized LCA results 5
S2.2 Normalized LCA results for each scenario 7

Bibliography **7**

S1. Introduction

S1.1 Life Cycle Assessment on composites

Table S1: *LCA studies on composites. C-LCA=comparative LCA; A-LCA=Attributional LCA.*

Ref.	Materials	Application	Type of LCA	Impact assessment	System Boundaries	EoL
[1]	Steel and CFRP	Automotive	C-LCA	Eco-Indicator 99 aggregation	cradle to grave	Incineration
[2]	Steel and CFRP	Automotive	C-LCA and life cycle energy	Not clear	cradle to grave	Thermal recycling
[3]	CFRP waste	Not specified	C-LCA	impact 2002+	EoL	Pyrolysis, Incineration with energy recovery and landfilling
[4]	DL-PU panel, ABA-Cotton panel, DL-cotton panel	Automotive	C-LCA	CML 2002	cradle to grave	Landfill and incineration with energy recovery
[5]	Lightweight materials	Automotive	C-LCA	CML-IA	cradle to grave	Incineration, open-loop recycling and closed-loop recycling
[6]	CFRP	Not specified	A-LCA	CML 2000	cradle to grave	Chemical recycling
[7]	CFRP waste	Automotive	C-LCA	Not clear	EoL	Landfill, incineration and mechanical recycling
[8]	Composite waste	Not specified	C-LCA	EPD	EoL	Chemical and mechanical recycling
[9]	Aluminum alloy and CFRP	aviation	C-LCA	Eco-Indicator 99 aggregation	cradle to gate + EoL	Thermal treament (pyrolysis)
[10]	CFRP waste	Not specified	C-LCA	Not clear	EoL	Landfill, incineration, mechanical recycling, pyrolysis recycling, fluidized bed recycling, and chemical recycling
[11]	Biopolymer derived from FDCA	Not specified	environmental assessment	ReCiPe mid-point	cradle to gate	NA
[12]	Glass fiber composite and hybrid composite	Automotive	C-LCA	US EPA TRACI2.1	cradle to grave	Landfill
[13]	CFRP waste	Not specified	C-LCA	CML-IA; ILCD 2011 midpoint	EoL + cradle-to-grave (two cycles)	Thermolysis recycling and landfill +incineration
[14]	Kenaf and glass fiber reinforced cement	Construction	C-LCA	BEES	cradle to grave	Landfill
[15]	Polyurethane and rockwool filled composite	Construction	C-LCA	CML-IA	cradle to grave	Dismantle of material, reuse, recycling and storage
[16]	Miscanthus carbon based composite	Automotive	C-LCA	US EPA TRACI2.1	cradle to grave	Landfill

S2. Results

The following section provides additional information on the analyses shown in the main paper. In particular, it provides the normalized LCA results according to the normalization factor by [17].

S2.1 Normalized LCA results

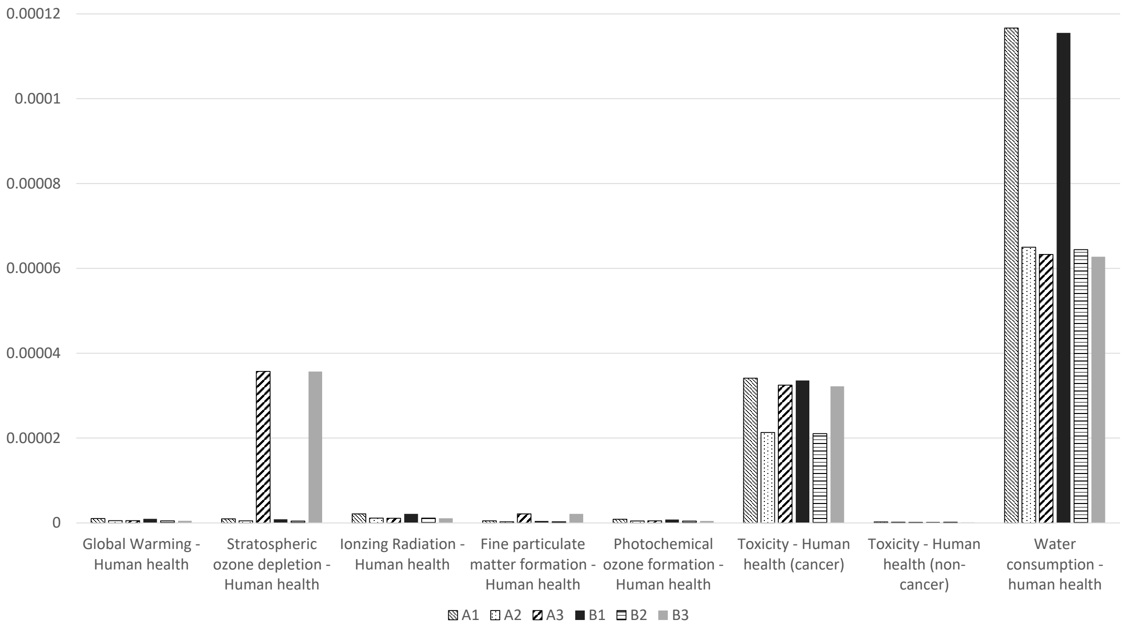

Figure S1: Normalization of LCA results of the six scenarios - human health.

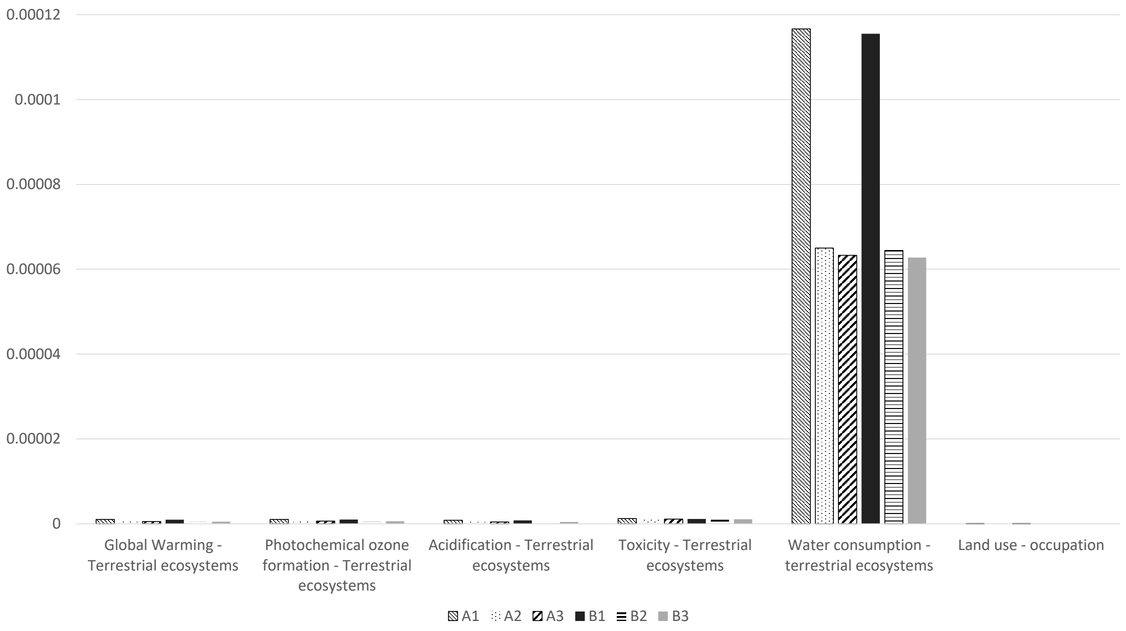

Figure S2: Normalization of LCA results of the six scenarios - terrestrial ecosystem.

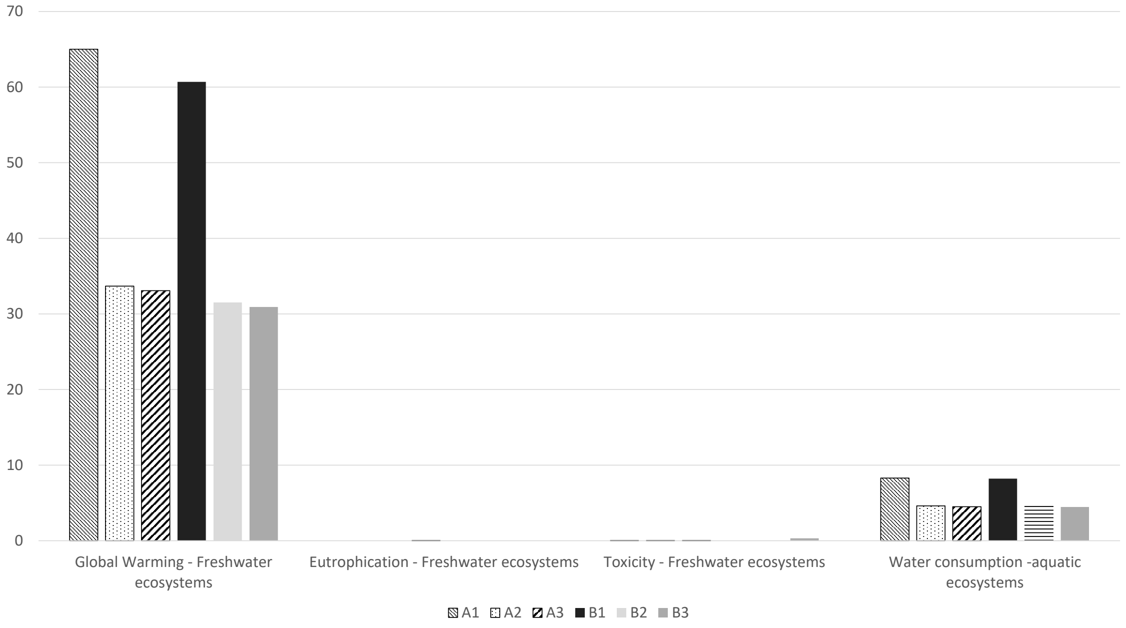

Figure S3: Normalization of LCA results of the six scenarios - freshwater ecosystem.

S2.2 Normalized LCA results for each scenario

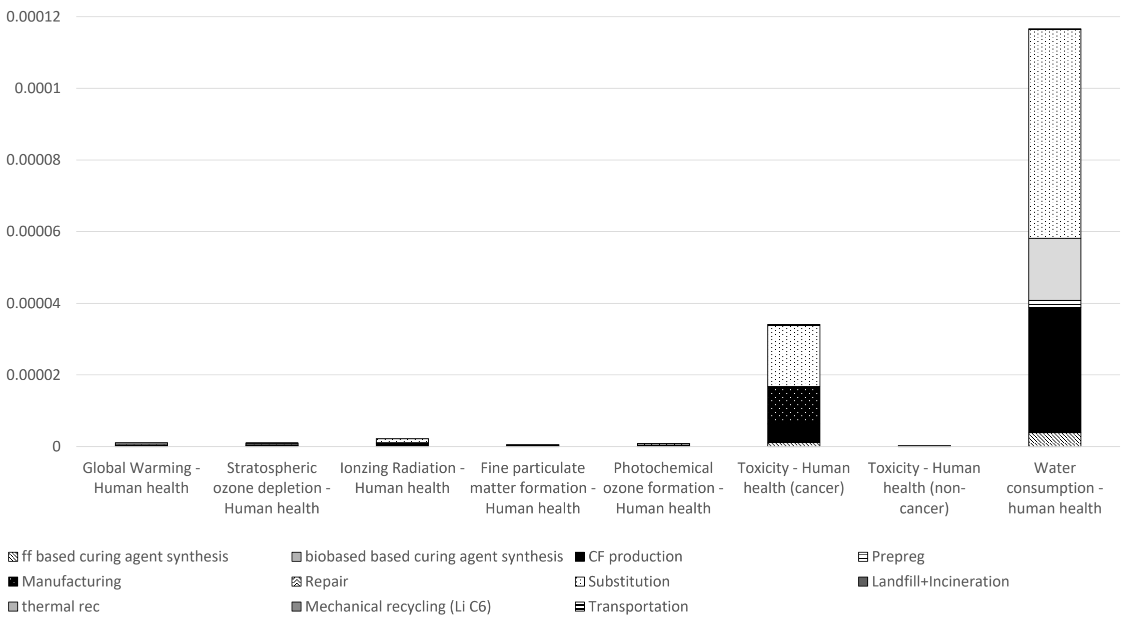

Figure S4: Normalization of LCA results of scenario A1. Water consumption and Toxicity are the two categories with higher environmental impact. CF production, manufacturing and substitution are the steps with highest contribution.

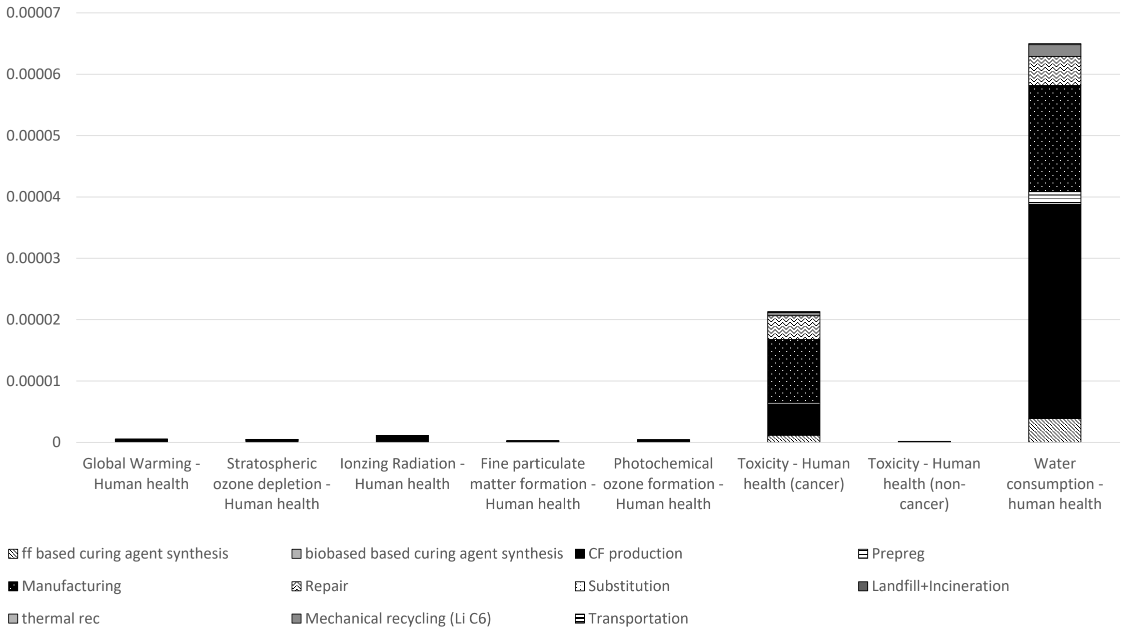

Figure S5: Normalization of LCA results of scenario A2. Water consumption and Toxicity are the two categories with higher environmental impact. CF production and manufacturing are the steps with highest contribution

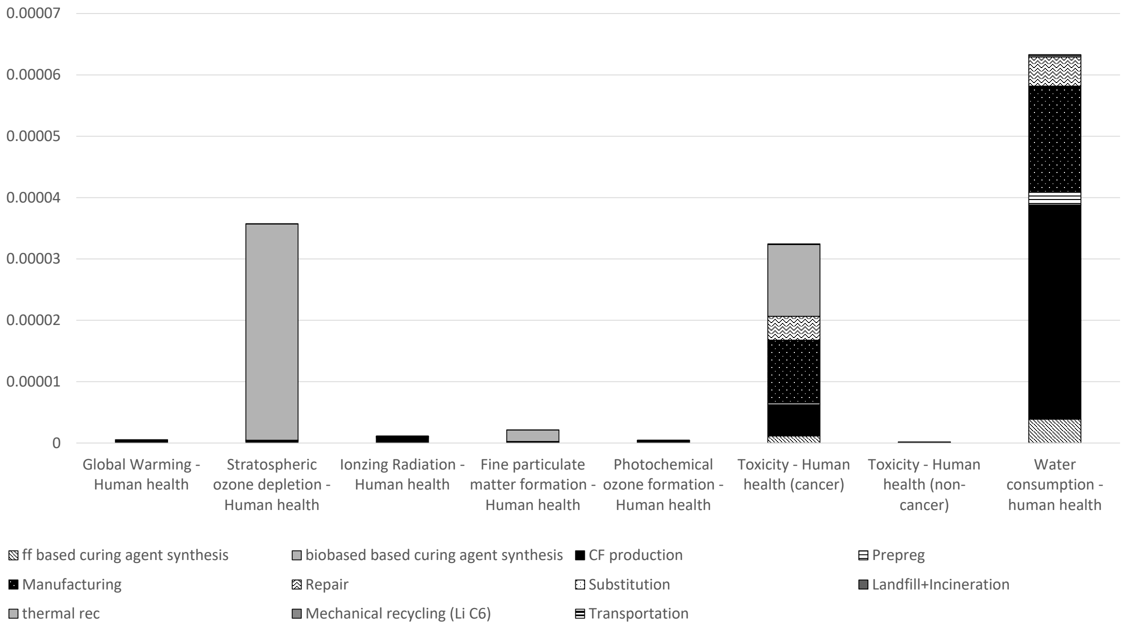

Figure S6: Normalization of LCA results of scenario A3. Stratospheric ozone depletion,Water consumption and Toxicity are the two categories with higher environmental impact. CF production, manufacturing and thermal recycling are the steps with highest contribution

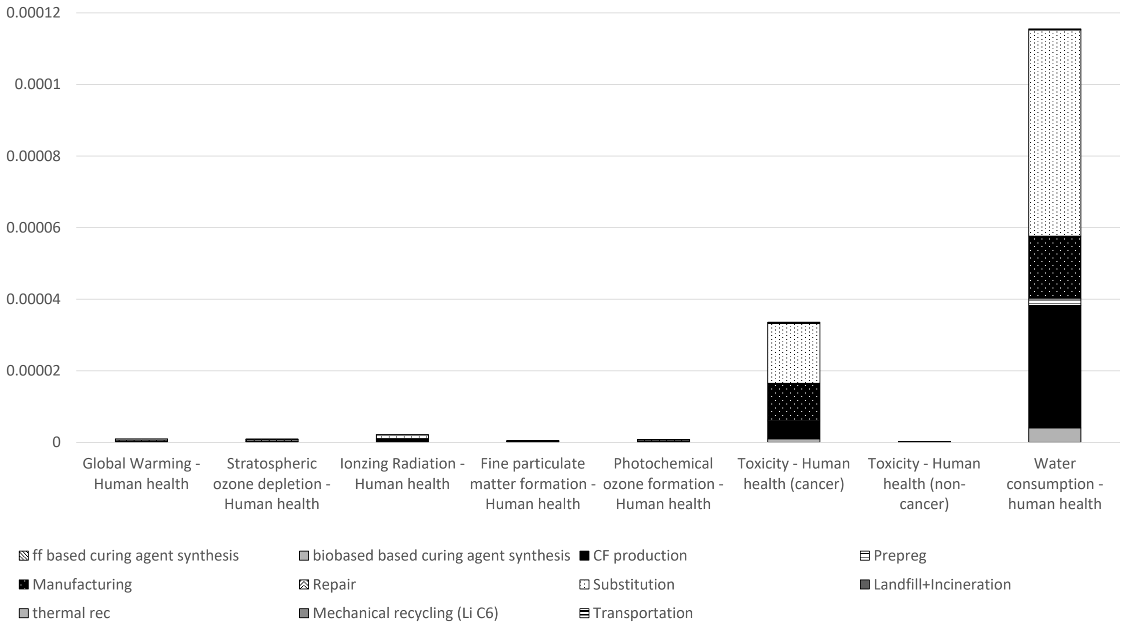

Figure S7: Normalization of LCA results of scenario B1. Water consumption and Toxicity are the two categories with higher environmental impact. CF production, manufacturing and substitution are the steps with highest contribution.

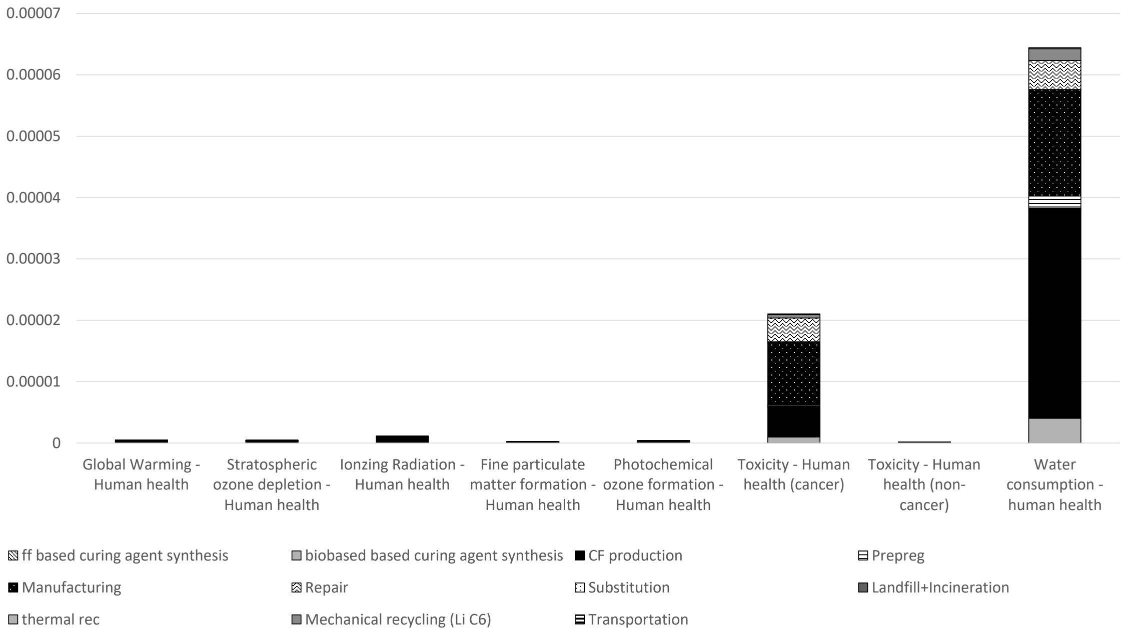

Figure S8: Normalization of LCA results of scenario A2. Water consumption and Toxicity are the two categories with higher environmental impact. CF production and manufacturing are the steps with highest contribution

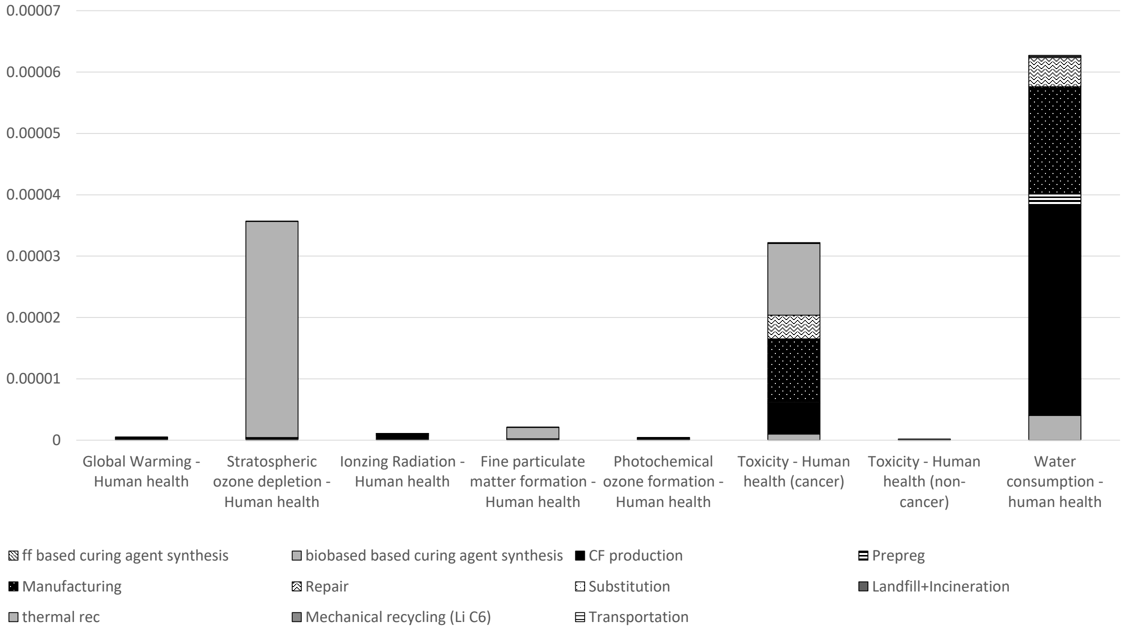

Figure S9: Normalization of LCA results of scenario A3. Stratospheric ozone depletion,Water consumption and Toxicity are the two categories with higher environmental impact. CF production, manufacturing and thermal recycling are the steps with highest contribution

Bibliography

[1] J. R. Duflou, J. De Moor, I. Verpoest, and W. Dewulf, "Environmental impact analysis of composite use in car manufacturing," *CIRP Annals - Manufacturing Technology*, vol. 58, no. 1, pp. 9–12, 2009.

[2] S. Das, "Life cycle assessment of carbon fiber-reinforced polymer composites," *International Journal of Life Cycle Assessment*, vol. 16, no. 3, pp. 268–282, 2011.

[3] R. A. Witik, R. Teuscher, V. Michaud, C. Ludwig, and J. A. E. Månson, "Carbon fibre reinforced composite waste: An environmental assessment of recycling, energy recovery and landfilling," *Composites Part A: Applied Science and Manufacturing*, vol. 49, pp. 89–99, 2013. [Online]. Available: http://dx.doi.org/10.1016/j.compositesa.2013.02.009

[4] T. D. S. Pegoretti, F. Mathieux, D. Evrard, D. Brissaud, and J. R. D. F. Arruda, "Use of recycled natural fibres in industrial products: A comparative LCA case study on acoustic components in the Brazilian automotive sector," *Resources, Conservation and Recycling*, vol. 84, pp. 1–14, 2014.

[5] M. Raugei, D. Morrey, A. Hutchinson, and P. Winfield, "A coherent life cycle assessment of a range of lightweighting strategies for compact vehicles," *Journal of Cleaner Production*, vol. 108, pp. 1168–1176, 2015.

[6] A. D. La Rosa, D. R. Banatao, S. J. Pastine, A. Latteri, and G. Cicala, "Recycling treatment of carbon fibre/epoxy composites: Materials recovery and characterization and environmental impacts through life cycle assessment," *Composites Part B: Engineering*, vol. 104, pp. 17–25, 2016. [Online]. Available: http://dx.doi.org/10.1016/j.compositesb.2016.08.015

[7] X. Li, R. Bai, and J. McKechnie, "Environmental and financial performance of mechanical recycling of carbon fibre reinforced polymers and comparison with conventional disposal routes," *Journal of Cleaner Production*, vol. 127, no. 2016, pp. 451–460, 2016. [Online]. Available: http://dx.doi.org/10.1016/j.jclepro.2016.03.139

[8] N. A. Shuaib and P. T. Mativenga, "Carbon Footprint Analysis of Fibre Reinforced Composite Recycling Processes," *Procedia Manufacturing*, vol. 7, pp. 183–190, 2017.

[9] Y. F. Khalil, "Eco-efficient lightweight carbon-fiber reinforced polymer for environmentally greener commercial aviation industry," *Sustainable Production and Consumption*, vol. 12, no. May 2016, pp. 16–26, 2017. [Online]. Available: http://dx.doi.org/10.1016/j.spc.2017.05.004

[10] F. Meng, E. A. Olivetti, Y. Zhao, J. C. Chang, S. J. Pickering, and J. McKechnie, "Comparing Life Cycle Energy and Global Warming Potential of Carbon Fiber Composite Recycling Technologies and Waste Management Options," *ACS Sustainable Chemistry and Engineering*, vol. 6, no. 8, pp. 9854–9865, 2018.

[11] C. Isola, H. L. Sieverding, R. Raghunathan, M. P. Sibi, D. C. Webster, J. Sivaguru, and J. J. Stone, "Life cycle assessment of photodegradable polymeric material derived from renewable bioresources," *Journal of Cleaner Production*, vol. 142, pp. 2935–2944, 2017. [Online]. Available: http://dx.doi.org/10.1016/j.jclepro.2016.10.177

[12] M. Akhshik, S. Panthapulakkal, J. Tjong, and M. Sain, "Life cycle assessment and cost analysis of hybrid fiber-reinforced engine beauty cover in comparison with glass fiber-reinforced counterpart," *Environmental Impact Assessment Review*, vol. 65, no. September 2016, pp. 111–117, 2017. [Online]. Available: http://dx.doi.org/10.1016/j.eiar.2017.04.005

[13] A. O. Nunes, L. R. Viana, P. M. Guineheuc, V. A. da Silva Moris, J. M. F. de Paiva, R. Barna, and Y. Soudais, "Life cycle assessment of a steam thermolysis process to recover carbon fibers from carbon fiber-reinforced polymer waste," *International Journal of Life Cycle Assessment*, vol. 23, no. 9, pp. 1825–1838, 2018.

[14] C. Zhou, S. Q. Shi, Z. Chen, L. Cai, and L. Smith, "Comparative environmental life cycle assessment of fiber reinforced cement panel between kenaf and glass fibers," *Journal of Cleaner Production*, vol. 200, pp. 196–204, 2018. [Online]. Available: https://doi.org/10.1016/j.jclepro.2018.07.200

[15] E. Yılmaz, H. Arslan, and A. Bideci, "Environmental performance analysis of insulated composite facade panels using life cycle assessment (LCA)," *Construction and Building Materials*, vol. 202, pp. 806–813, 2019.

[16] P. Roy, F. Defersha, A. Rodriguez-Uribe, M. Misra, and A. K. Mohanty, "Evaluation of the life cycle of an automotive component produced from biocomposite," *Journal of Cleaner Production*, vol. 273, p. 123051, 2020. [Online]. Available: https://doi.org/10.1016/j.jclepro.2020.123051

[17] National Institute for Public Health and Environment and Ministry of Health Welfare and sport, "Normalization scores ReCiPe 2016," 2020. [Online]. Available: https://www.rivm.nl/en/documenten/normalization-scores-recipe-2016

List of Figures

S1 Normalization of LCA results of the six scenarios - human health. . . . 5
S2 Normalization of LCA results of the six scenarios - terrestrial ecosystem. 6
S3 Normalization of LCA results of the six scenarios - freshwater ecosystem. 6
S4 Normalization of LCA results of scenario A1. *Water consumption* and *Toxicity* are the two categories with higher environmental impact. *CF production*, *manufacturing* and *substitution* are the steps with highest contribution. 7
S5 Normalization of LCA results of scenario A2. *Water consumption* and *Toxicity* are the two categories with higher environmental impact. *CF production* and *manufacturing* are the steps with highest contribution . 8
S6 Normalization of LCA results of scenario A3. *Stratospheric ozone depletion*, *Water consumption* and *Toxicity* are the two categories with higher environmental impact. *CF production*, *manufacturing* and *thermal recycling* are the steps with highest contribution 8
S7 Normalization of LCA results of scenario B1. *Water consumption* and *Toxicity* are the two categories with higher environmental impact. *CF production*, *manufacturing* and *substitution* are the steps with highest contribution. 9
S8 Normalization of LCA results of scenario A2. *Water consumption* and *Toxicity* are the two categories with higher environmental impact. *CF production* and *manufacturing* are the steps with highest contribution . 9
S9 Normalization of LCA results of scenario A3. *Stratospheric ozone depletion*, *Water consumption* and *Toxicity* are the two categories with higher environmental impact. *CF production*, *manufacturing* and *thermal recycling* are the steps with highest contribution 10

List of Tables

S1 LCA studies on composites. C-LCA=comparative LCA; A-LCA=Attributional LCA. 4
